# Biliodigestive Anastomose – Technik und Outcome

**DOI:** 10.1007/s00104-025-02372-2

**Published:** 2025-09-09

**Authors:** Maximilian Brunner, Robert Grützmann

**Affiliations:** 1 Klink für Allgemein- und Viszeralchirurgie, Universitätsklinikum der Friedrich-Alexander-Universität Erlangen, Erlangen, Deutschland; 2 Krankenhausstr. 12, 91054 Erlangen, Deutschland

**Keywords:** Hepatopankreatobiliäre Chirurgie, Hepatikojejunostomie, Nahttechnik, Insuffizienz, Stenose, Hepatopancreatobiliary surgery, Hepaticojejunostomy, Suture technique, Insufficiency, Stenosis

## Abstract

Die biliodigestive Anastomose stellt ein zentrales Element der hepatopankreatobiliären Chirurgie dar. Sie erfordert eine präzise chirurgische Technik sowie detaillierte Kenntnisse der biliären Anatomie und Gefäßversorgung. Für die erfolgreiche Anlage sind eine spannungsfreie Naht, eine suffiziente Durchblutung der anastomosierten Strukturen und eine exakte Mukosa-zu-Mukosa-Adaptation unter Verwendung von feinem, resorbierbarem Nahtmaterial entscheidend. Die Hepatikojejunostomie hat sich als Goldstandard der biliodigestiven Anastomose etabliert. Derzeit vorliegende Studien zeigen keinen signifikanten Unterschied in der Morbidität zwischen offener und minimal-invasiver Technik, wobei Letztere zunehmend an Bedeutung gewinnt. Auch in Bezug auf die Nahttechnik (Einzelknopf- vs. fortlaufende Naht) zeigen sich keine Unterschiede in den klinischen Ergebnissen; allerdings ist die fortlaufende Nahttechnik mit einer Verkürzung der Anlagezeit und einer Reduktion der Kosten assoziiert. Komplikationen nach biliodigestiver Anastomose – insbesondere Insuffizienzen und Stenosen – sind klinisch relevant und beeinflussen Morbidität und Mortalität maßgeblich. Viele dieser Komplikationen lassen sich heutzutage jedoch mittels interventioneller Verfahren effektiv behandeln.

## Abstract (en)

The biliodigestive anastomosis represents a core element of hepatopancreatobiliary surgery. It requires a precise surgical technique and detailed knowledge of biliary anatomy and vascular supply. A tension-free suture, sufficient perfusion of the anastomosed structures and an exact mucosa-to-mucosa adaptation using delicate absorbable suture material are decisive for a successful construction. Hepaticojejunostomy has become established as the gold standard for a biliodigestive anastomosis. Currently available studies show no significant differences in morbidity between open and minimally invasive techniques, whereby the latter is becoming increasingly more important. There was also no difference in the clinical results with respect to the suture technique (interrupted vs. continuous suture); however, the continuous suture technique is associated with a shortening of the placement time and a reduction in costs. Complications after biliodigestive anastomosis, such as insufficiency and stenosis, are clinically relevant and substantially influence the morbidity and mortality; however, many of these complications can nowadays be effectively treated by interventional procedures.

Die biliodigestive Anastomose ist ein zentrales Verfahren der hepatopankreatobiliären Chirurgie und ein essenzieller Bestandteil bei operativen Eingriffen an Pankreas, Leber und Gallenwegen. Typische Indikationen sind maligne Erkrankungen wie Pankreas- oder Gallengangskarzinome sowie Lebermalignome, aber auch chronische Pankreatitiden, iatrogene Gallengangsverletzungen und komplexe benigne Strikturen. Ziel der biliodigestiven Anastomose ist die Herstellung einer dauerhaften, sicheren und komplikationsarmen biliären Drainage, um Folgeerkrankungen wie Cholestase, Cholangitis oder Leberfunktionsstörungen zu verhindern.

Trotz ihrer weiten Verbreitung bleibt die Anlage einer biliodigestiven Anastomose technisch anspruchsvoll, und die angewandten Techniken sind unter den Chirurgen durchaus sehr heterogen [1]. Anastomoseninsuffizienzen, Stenosen oder rezidivierende Infektionen stellen relevante postoperative Komplikationen dar, die die Lebensqualität der Patienten deutlich beeinträchtigen können.

In den letzten Jahren wurde die Evidenzlage zur operativen Technik und zu präventiven Maßnahmen kontinuierlich erweitert. Diese Arbeit gibt einen Überblick über die aktuelle Studienlage zur optimalen Durchführung der biliodigestiven Anastomose und beleuchtet die entscheidenden Einflussfaktoren auf das postoperative Ergebnis. Neben technischen Aspekten werden auch Strategien zur Vermeidung typischer Komplikationen dargestellt.

## Chirurgische Techniken

**Abb. 1:**
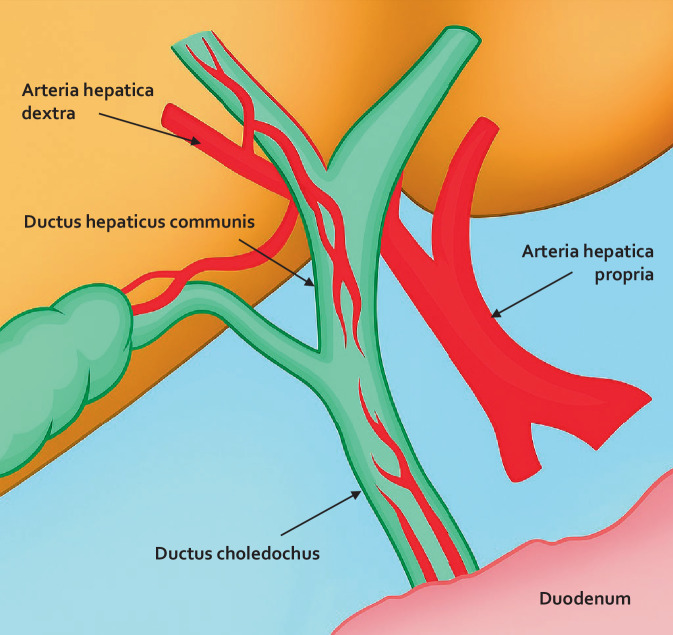
Arterielle Gefäßversorgung des Ductus hepaticus communis (*DHC*) und Ductus choledochus. (Mod. nach [2])

Die Basis für eine erfolgreiche biliodigestive Anastomose ist zum einen die präzise Kenntnis der Anatomie der Gallenwege und ihrer Variationen, zum anderen die konsequente Einhaltung grundlegender chirurgischer Prinzipien der Anastomosierung. Zusätzlich spielen spezifische Aspekte der Gallenwegschirurgie eine zentrale Rolle [2]:

- Spannungsfreiheit: Mit an oberster Stelle steht die spannungsfreie Anlage der Anastomose. Nur so kann eine sichere Heilung gewährleistet und das Risiko von Nahtinsuffizienzen minimiert werden.
- Optimale Durchblutung: Ebenso entscheidend ist eine gute Durchblutung der anastomosierten Strukturen – sowohl des Gallengangs als auch des Dünndarms. Hierbei ist die Kenntnis der Gefäßversorgung des extrahepatischen Gallenwegssystems von essenzieller Bedeutung. Während das proximale Gallenwegssystem, also die Ductus hepaticus dextra und sinistra sowie der Ductus hepaticus communis (DHC), von proximal aus der A. hepatica dextra versorgt werden, wird das distale Gallenwegssystem, also der Ductus choledochus, insbesondere vom Duodenum bzw. Pankreaskopf her versorgt (Abb. 1). Daher stellen Kompromittierungen bzw. Verletzungen der A. hepatica dextra, welche nicht selten im Rahmen von Gallenwegsverletzungen simultan einhergehen, ein besonderes Problem dar, da eine biliodigestive Anastomose in diesen Fällen mit einer signifikant höheren Komplikationsrate, insbesondere Insuffizienzrate, assoziiert ist [3]. Des Weiteren impliziert die dargestellte Gefäßversorgung des Gallenwegssystems, dass die biliodigestive Anastomose optimalerweise an einem kurzen DHC-Stumpf erfolgen sollte, da hier die Durchblutung des DHCs am besten ist. Ziel ist es, die Anastomose proximal der Einmündung des Ductus cysticus und etwa 1–2 cm distal der Ductus-hepaticus-Gabelung anzulegen. Bezüglich der hochgezogenen Dünndarmschlinge ist ebenso auf eine adäquate Durchblutung zu achten.
- Exakte Mukosa-Mukosa-Adaptation: Für eine dichte und funktionelle Verbindung ist die präzise Anpassung der Schleimhautränder entscheidend. Dabei ist eine atraumatische Nahttechnik unerlässlich, um Gewebequetschungen zu vermeiden.
- Feines Nahtmaterial: Zum Einsatz kommt feines, monofiles, resorbierbares Nahtmaterial, üblicherweise 5‑0 oder 6‑0. Beim minimal-invasiven Vorgehen kommen auch häufig Fäden mit Widerhaken zum Einsatz, da sie das Halten der Nahtspannung erleichtern.

Die sorgfältige Einhaltung dieser Prinzipien ist entscheidend für den langfristigen Erfolg der biliären Rekonstruktion.

### Technik der Hepatikojejunostomie

**Abb. 2:**
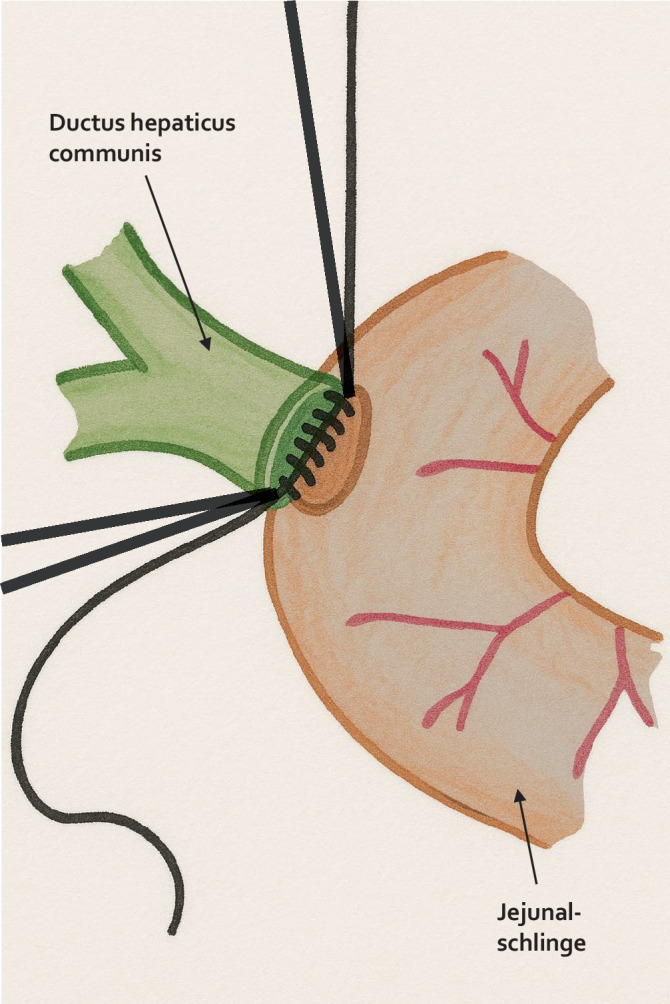
Schematische Darstellung einer Hepatikojejunostomie. Der Ductus hepaticus communis ist mit 2 Haltfäden aufgespannt. Die Hinterwandnahtreihe wurde fortlaufend angelegt

**Abb. 3:**
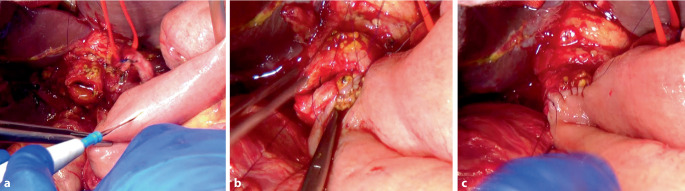
Intraoperative Bilder einer Hepatikojejunostomie in fortlaufender Nahttechnik. **a** Der Ductus hepaticus communis (DHC) ist mit Haltfäden aufgehängt. Die hochgezogene Jejunalschlinge wird mittels Diathermie inzidiert. **b** Anschließend wird die Hinterwandnahtreihe mittels fortlaufender Naht durchgeführt und zuletzt mit dem Eckfaden der Gegenseite verknotet. **c** Anschließend wird die Vorderwandnahtreihe durchgeführt, indem aus beiden Ecken heraus die Vorderwandnaht gestochen wird und schließlich die Fäden verknotet werden

**Abb. 4:**
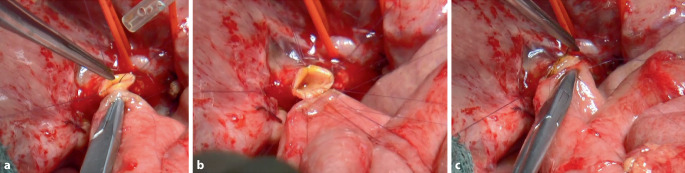
Intraoperative Bilder einer Hepatikojejunostomie in Einzelknopfnahttechnik. **a** Der Ductus hepaticus communis (DHC) ist mit Haltfäden aufgehängt. Die Hinterwandnähte werden einzeln vorgelegt. **b** Nach komplettem Vorlegen der Hinterwandnähte werden diese geknotet, und so wird die Hinterwandnahtreihe komplettiert. **c** Anschließend wird die Vorderwandnahtreihe durchgeführt, indem auch hier die Nähte vorgelegt und schließlich verknotet werden

Nach präziser Darstellung und Mobilisation des Ductus hepaticus communis wird dieser zunächst mit 2 Eckfäden (z. B. 5‑0 PDS) aufgespannt, um die Anastomosenfläche zu exponieren und eine gute Übersicht zu schaffen (Abb. 2). Anschließend erfolgt die Anastomosierung zur hochgezogenen inzidierten jejunalen Schlinge (Abb. 3a) z. B. in folgender Reihenfolge:

- Hinterwandnaht: Beginn an einer der beiden Ecken mit einer kontinuierlichen (Abb. 3b) oder Einzelknopfnaht (Abb. 4a, b) (je nach Technik), fortlaufend bis zur gegenüberliegenden Ecke. Dabei erfolgt eine exakte Mukosa-Mukosa-Adaptation.
- Fixierung der Eckfäden: Beide Eckfäden werden nun gestochen und unter Sicht verknotet. Bei fortlaufender Nahttechnik wird der Hinterwandfaden mit dem entsprechenden Eckfaden verknotet.
- Vorderwandnaht: Von der ersten Ecke werden einige Nähte der Vorderwand gesetzt, dann wird der zweite Eckfaden gestochen und die restliche Vorderwandnaht bis zum Ausgangspunkt fertiggestellt (Abb. 3c und 4c).
- Schlussknoten: Am Ende erfolgt der finale Knoten der Vorderwandnaht.
- Dichtigkeitsprüfung: Abschließend wird ein Weißkompressentest durchgeführt (Einlegen einer trockenen Kompresse zur Prüfung auf Galleleckage).

Eine standardisierte Technik gewährleistet Übersicht, Sicherheit und eine möglichst spannungsfreie, durchblutete Anastomose. Die Verwendung einer Lupenbrille kann insbesondere bei sehr kleinen Gallengängen helfen, die Nähte präzise zu setzen und somit die Anastomosenqualität zu optimieren.

### Durchmesser des Gallengangs

**Abb. 5:**
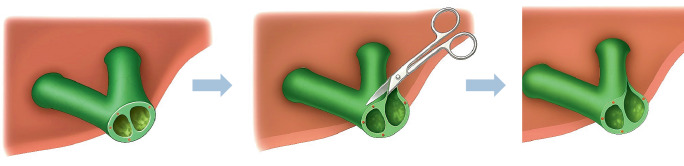
Erweiterungsplastik nach Hepp-Couinaud

Die Komplexität der biliodigestiven Anastomose wird zusätzlich vom Durchmesser des zu anastomosierenden Gallengangs bestimmt. Besonders kleine Gallengänge, etwa Segmentgallengänge, erhöhen die technische Herausforderung und erfordern oft spezielle Techniken. Da der Durchmesser des Ductus hepaticus nahe der Bifurkation meist am größten ist, bestätigt dies erneut die Vorteile einer Anastomose in diesem Bereich hinsichtlich Durchblutung und Handhabbarkeit. Ist der Gallengang dennoch zu eng, können Erweiterungsplastiken wie die nach Hepp-Couinaud angewendet werden, bei der der linke Ductus hepaticus ventral eingeschnitten wird, um den Durchmesser zu vergrößern und somit die Sicherheit der Anastomose zu erhöhen (Abb. 5).

### Art der biliodigestiven Anastomose

Im Verlauf der chirurgischen Entwicklung wurden zahlreiche Varianten der biliodigestiven Anastomose beschrieben. Heute gilt die Hepatikojejunostomie als etablierter Standard in der chirurgischen Rekonstruktion der Gallenwege.

Als mögliche Alternative wurde insbesondere die Choledochoduodenostomie, also die direkte Anastomosierung des Ductus choledochus oder Ductus hepaticus communis an das Duodenum, diskutiert. Befürworter führten eine vermeintlich physiologischere Rekonstruktion und die endoskopische Erreichbarkeit mittels retrograder Cholangiographie (ERC) als Vorteile an – auch wenn für die „Physiologie“ dieser Rekonstruktion bislang keine evidenzbasierten Belege vorliegen. Vergleichende randomisierte kontrollierte Studien zwischen Hepatikojejunostomie und Choledochoduodenostomie existieren nicht.

In einer retrospektiven, Propensity-Score-gematchten Analyse von Schreuder et al. an 52 Patienten zeigte sich zwar kein Unterschied in den kurzfristigen postoperativen Komplikationen, jedoch war die Choledochoduodenostomie im Langzeitverlauf mit signifikant höheren Raten an rezidivierenden Cholangitiden und chirurgischen Reinterventionen assoziiert [4]. Zusätzlich sprechen anatomische Aspekte gegen die Choledochoduodenostomie: Die Durchblutung ist im proximalen Ductus hepaticus communis günstiger, während die fixe Lage des Duodenums häufiger zu Anastomosenspannung führt. Zusammengenommen bestätigt dies die Überlegenheit der Hepatikojejunostomie in Bezug auf Funktionalität und Langzeitstabilität.

Als weitere, jedoch sehr selektiv diskutierte Alternative wurde in Einzelfällen bei kurzstreckigen Gallengangsverletzungen die biliobiliäre Anastomose (z. B. End-zu-End-Anastomose) versucht. Die Datenlage hierzu ist jedoch eindeutig: Retrospektive Studien zeigen Insuffizienzraten von bis zu 20 % und Stenoseraten von 70–80 %, was diese Technik in der Regel als inadäquat erscheinen lässt [5].

### Einzelknopfnahtechnik versus fortlaufende Nahttechnik

Eine zentrale technische Fragestellung bei der Anlage einer biliodigestiven Anastomose betrifft die Wahl zwischen Einzelknopfnaht- und fortlaufender Nahttechnik. Während sich frühere Erkenntnisse hierzu weitgehend auf Daten aus der Transplantationschirurgie stützten – einem Bereich mit erhöhtem technischem Anspruch und spezieller Patientenselektion – ist deren Übertragbarkeit auf elektive nicht-transplantationschirurgische Eingriffe nur eingeschränkt möglich.

In den letzten Jahren hat sich die Studienlage jedoch durch mehrere Arbeiten, darunter auch randomisiert kontrollierte Studien, deutlich verbessert. Einen Überblick über die aktuelle Evidenz liefert Tab. 1. Insgesamt zeigen die verfügbaren Daten keinen signifikanten Unterschied zwischen Einzelknopf- und fortlaufender Nahttechnik hinsichtlich der Rate an Anastomoseninsuffizienzen, postoperativen Stenosen/Strikturen oder Cholangitiden [6–11].

**Tab. 1:** Vergleich der Studien zur chirurgischen Technik: Einzelknopfnahttechnik (EKN) vs. fortlaufende Nahttechnik (FN)

Autor	Jahr	Art der Studie	*n*	Insuffizienz (%)		Stenose/Striktur (%)		Cholangitis (%)		Kosten	Zeit (min)	
				EKN	FN	EKN	FN	EKN	FN	FN vs. EKN	EKN	FN
Tatsuguchi et al. [6]	2018	Retrospektiv	228	1,2	1,2	8,6	6,2	11,1	7,5	*−92* *%*	*27,0*	*16,2*
Saxena et al. [7]	2021	Retrospektiv	556	*10,2*	*6,8*	4,6	4,7	NA	NA	*−83* *%*	*44,6*	*21,4*
Natsume et al. [8]	2021	Retrospektiv	172	0	0	*1,8*	*15,6*	0	6,0	NA	NA	NA
Seifert et al. [9]	2022	RCT	82	10,3	14,6	0	0	0	2,4	NA	*12,0*	*22,4*
Yadav et al. [10]	2024	Retrospektiv	34	11,1	6,2	0	0	NA	NA	*−75* *%*	*38,6*	*16,2*
Brunner et al. [11]	2024	RCT	100	2,0	2,0	0	0	NA	NA	*−68* *%*	*17,5*	*11,6*

*NA* nicht angegeben

Kursiv gedruckt = signifikanter Unterschied

Vorteile der fortlaufenden Nahttechnik zeigen sich jedoch insbesondere in operativen Aspekten: Mehrere Studien berichten über eine signifikant kürzere Anlagezeit sowie potenzielle Kosteneinsparungen durch den geringeren Verbrauch an Nahtmaterial und Instrumenten [6, 7, 10, 11]. Bei sehr kleinen Gallengängen < 5 mm bietet die Einzelknopfnahttechnik allerdings manchmal eine bessere Übersichtlichkeit.

### Einreihig versus zweireihig

Bezüglich der technischen Ausführung der biliodigestiven Anastomose – insbesondere der Frage nach einreihiger vs. zweireihiger Nahttechnik – existieren bislang keine direkten vergleichenden Studien. Ein Blick in die Literatur zeigt jedoch, dass die einreihige Anastomosentechnik derzeit die mit Abstand am häufigsten angewandte Methode darstellt.

Vereinzelt berichten Studien auch über die Anwendung der zweireihigen Technik mit vielversprechenden Ergebnissen. So publizierten Napoli et al. ihre Erfahrungen mit einer zweireihigen, fortlaufenden Nahttechnik im Rahmen von 800 Pankreaskopfresektionen. Die Autoren dokumentierten eine bemerkenswert niedrige Anastomoseninsuffizienzrate von lediglich 0,6 %. Auch die Langzeitergebnisse mit Stenoserate (6,1 %), Cholangitiden (5,4 %) und Reinterventionen (2,0 %) nach über 3 Jahren Follow-up waren überzeugend [12].

Eine weitere Arbeit von Takagi et al. untersuchte eine zweireihige Einzelknopfnahttechnik der Hepatikojejunostomie im Rahmen roboterassistierter Pankreaskopfresektionen. In dieser kleineren Kohorte von 42 Patienten wurden eine Insuffizienzrate von 2,4 % und eine Stenoserate von ebenfalls 2,4 % im 1‑jährigen Follow-up berichtet [13].

Zusammenfassend lässt sich feststellen, dass die zweireihige Nahttechnik – obwohl seltener angewendet – in einzelnen Arbeiten gute Resultate erzielt hat. Aufgrund des Fehlens prospektiv randomisierter Vergleichsstudien kann derzeit jedoch keine abschließende Empfehlung zur Überlegenheit einer der beiden Techniken gegeben werden. Aktuell stellt die einreihige Nahttechnik die in den meisten Kliniken bevorzugte Methode dar.

### Minimal-invasiv oder offen

Zum direkten Vergleich der offenen mit der minimal-invasiven Anlage einer Hepatikojejunostomie existieren bislang keine randomisiert kontrollierten Studien. Da die Hepatikojejunostomie jedoch ein zentraler Bestandteil jeder Pankreaskopfresektion ist, können die in diesem Kontext durchgeführten RCTs zum Vergleich von offenen und minimal-invasiven Pankreaskopfresektionen zumindest zur Beurteilung der Anastomoseninsuffizienzraten herangezogen werden.

Die Tab. 2 gibt einen Überblick über die derzeitige Datenlage. In sämtlichen verfügbaren randomisiert kontrollierten Studien zur Pankreaskopfresektion zeigten sich keine signifikanten Unterschiede in der Rate an Hepatikojejunostomieinsuffizienzen zwischen der offenen und der minimal-invasiven Technik [14–19]. Diese Ergebnisse werden durch eine aktuelle Metaanalyse gestützt, welche neben RCTs auch Propensity-Score-gematchte Studien zum Vergleich der offenen vs. roboterassistierten Pankreaskopfresektion einbezog. Die Insuffizienzraten waren hierbei nahezu identisch (robotisch 6,6 %, offen 6,5 %) [20].

**Tab. 2:** Insuffizienzraten der biliodigestiven Anastomose im Rahmen von randomisiert kontrollierten Studien zum offenen vs. minimal-invasiven Vorgehen zur Pankreaskopfresektion

Autor	Jahr	Art der Studie	*n*	Minimal-invasives Vorgehen	Biliäre Insuffizienzrate (%)		
					Offen	MI	*p*
Palanivelu et al. [14]	2017	Monozentrisch	64	Laparoskopisch	6,3	9,4	0,419
Poves et al. [15]	2018	Monozentrisch	61	Laparoskopisch	10,3	3,1	0,13
van Hilst et al. [16]	2019	Multizentrisch	99	Laparoskopisch	10	12	0,78
Wang et al. [17]	2021	Multizentrisch	594	Laparoskopisch	14	13	0,72
Liu et al. [19]	2024	Multizentrisch	161	Robotisch	23	16	0,30
Klotz et al. [18]	2024	Monozentrisch	62	Robotisch	9,1	17,2	0,339

Langzeitdaten zur Stenoserate nach minimal-invasiver Hepatikojejunostomie fehlen in den genannten Studien jedoch weitgehend. Eine retrospektive Untersuchung von Nagakawa et al. analysierte die Stenoserate bei 103 Patienten nach laparoskopischer Pankreaskopfresektion und berichtete nach einem medianen Follow-up von 4 Jahren eine Rate von 7,8 %, was vergleichbar mit Werten aus der offenen Chirurgie ist [21].

Weitere Erkenntnisse zur minimal-invasiven Anlage der Hepatikojejunostomie stammen aus Studien zur chirurgischen Therapie von Choledochuszysten. Die hierzu publizierten Fallserien berichten übereinstimmend über exzellente Ergebnisse und niedrige Komplikationsraten [22, 23].

Insgesamt lässt sich zusammenfassen, dass die minimal-invasive Anlage einer Hepatikojejunostomie unter erfahrenen Händen mindestens gleichwertige Ergebnisse wie die offene Technik liefert. Angesichts technischer Weiterentwicklungen und zunehmender Expertise ist davon auszugehen, dass der minimal-invasiven Herangehensweise künftig eine wachsende Bedeutung zukommt.

## Outcome

Die bedeutendsten Komplikationen nach Anlage einer biliodigestiven Anastomose sind im Frühverlauf v. a. die Anastomoseninsuffizienz, während im mittel- bis langfristigen Verlauf die Ausbildung einer Stenose oder Striktur im Vordergrund steht. Zusätzlich können rezidivierende Cholangitiden auftreten, die für die betroffenen Patientinnen und Patienten mit erheblicher Morbidität und Einschränkung der Lebensqualität verbunden sind.

Die klinische Relevanz dieser Komplikationen wurde eindrücklich durch Jester et al. belegt. In ihrer Analyse von 924 Patientinnen und Patienten nach Pankreaskopfresektion zeigte sich, dass eine Insuffizienz der Hepatikojejunostomie mit einem signifikanten Anstieg der Morbidität von 24 % auf 54 % sowie der Mortalität von 3,6 % auf 17 % assoziiert war. Eine isolierte Insuffizienz der Hepatikojejunostomie hatte einen größeren negativen Einfluss auf die postoperative Morbidität und Mortalität als eine alleinige Insuffizienz der Pankreasanastomose. Das gleichzeitige Auftreten beider Komplikationen potenzierte das Risiko und führte zu einer Mortalitätsrate von gar 32 % [24].

### Anastomoseninsuffizienz

Die Inzidenz von Anastomoseninsuffizienzen nach biliodigestiven Anastomosen zeigt in der Literatur eine erhebliche Spannbreite und liegt – je nach zugrunde liegender Grunderkrankung, Patientenkollektiv und operativem Vorgehen – zwischen 0,6 und 23 % [4–20, 25, 26]. Zahlreiche Studien haben Risikofaktoren identifiziert, die mit einem erhöhten Auftreten von Insuffizienzen assoziiert sind. Zu den präoperativen Faktoren zählen insbesondere das männliche Geschlecht, Übergewicht, eine vorangegangene Cholangitis sowie durchgeführte ERCPs. Ebenso wurden eine präoperative Radiochemotherapie sowie eine Hypoalbuminämie (< 3,5 g/dl) als relevante Prädiktoren beschrieben. Intraoperativ gelten ein komplexer Verlauf mit längerer Operationszeit, insbesondere bei simultaner Leberresektion, als zusätzliche Belastung. Auch technische Aspekte wie eine unzureichende Durchblutung des Ductus hepaticus oder des Jejunums sowie eine übermäßige Anastomosenspannung erhöhen das Risiko für eine Insuffizienz signifikant. Ergänzend besteht eine hohe Koinzidenz mit postoperativen Pankreasfisteln, die häufig sekundär zu einer gestörten Heilung der biliodigestiven Anastomose führen [25, 26].

Klinisch manifestiert sich eine Anastomoseninsuffizienz typischerweise durch eine gallige Sekretion über intraabdominelle Drainagen, Fieber oder erhöhte Entzündungsparameter im Labor. Die bildgebende Diagnostik, insbesondere die kontrastmittelgestützte Computertomographie, ist essenziell zur Detektion perianastomotischer Flüssigkeitsansammlungen. In vielen Fällen kann eine CT-gestützte Drainage sowohl diagnostisch als auch therapeutisch wertvoll sein.

**Abb. 6:**
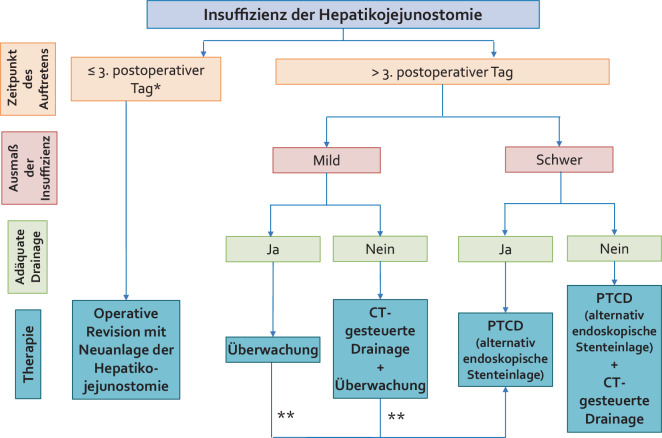
Therapiealgorithmus bei Insuffizienz der Hepatikojejunostomie. *PTCD* perkutane transhepatische Cholangiodrainage

**Abb. 7:**
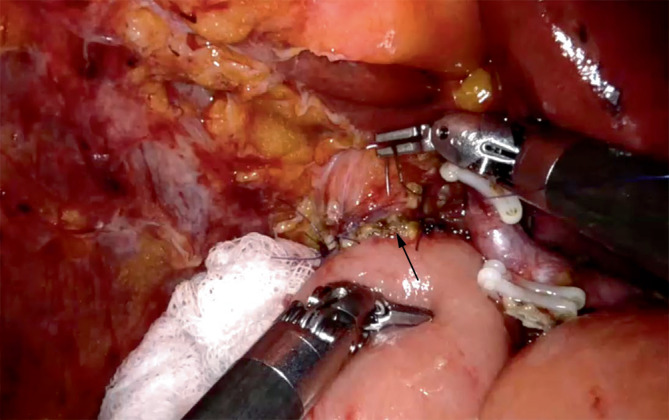
Robotische Revision bei früher punktförmiger Insuffizienz der Hepatikojejunostomie (*Pfeil*) nach robotischer Whipple-Operation

**Abb. 8:**
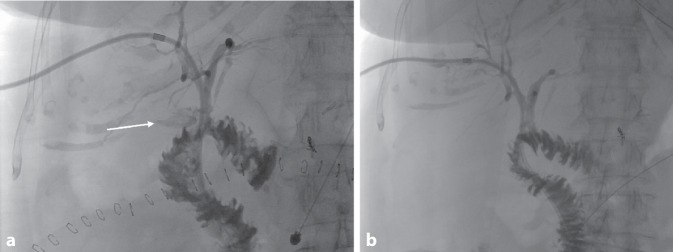
PTCD bei Insuffizienz der Hepatikojejunostomie. **a** Bei initialer Anlage zeigt sich eine Kontrastmittelfahne (*Pfeil*) als Nachweis der Insuffizienz. **b** Darstellung der PTCD vor geplanter Entfernung bei abgeheilter Insuffizienz

**Abb. 9:**
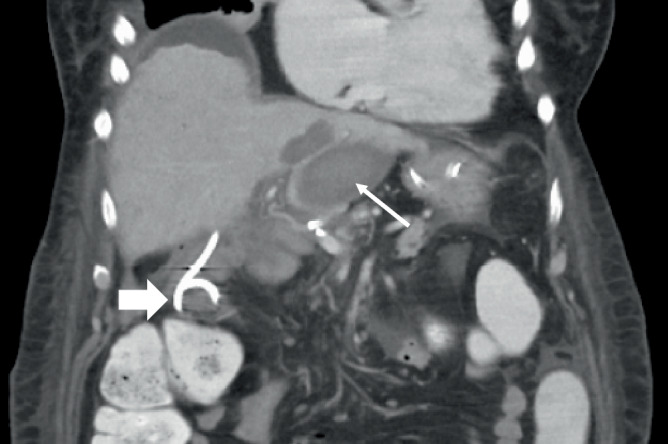
Subhepatisches drainagebedürftiges Biliom (*dünner Pfeil*) bei Insuffizienz der Hepatikojejunostomie mit bereits einliegender PTCD (*dicker Pfeil*)

Das therapeutische Vorgehen richtet sich maßgeblich nach dem Zeitpunkt des Auftretens sowie dem klinischen Zustand des Patienten (Abb. 6):

- Frühe Insuffizienzen (innerhalb der ersten 72 h postoperativ) sollten in der Regel chirurgisch revidiert werden, idealerweise mit einer Neuanlage der Hepatikojejunostomie (Abb. 7).
- Späte Anastomoseninsuffizienzen stellen eine Domäne der interventionellen Therapie dar. Hier kommt primär die perkutane transhepatische Cholangiographie mit Drainage (PTCD) zum Einsatz, welche sowohl die Galle ableitet als auch die Anastomose schient und so die Heilung unterstützt (Abb. 8). In ausgewählten Fällen mit gut zugänglicher Hepatikojejunostomie kann auch eine endoskopische Stenteinlage (via ERCP) erfolgen. Zudem sollten durch die Insuffizienz entstandene Verhalte bzw. Biliome adäquat z. B. CT-gesteuert drainiert werden (Abb. 9).
- Bei kleinen, lokal begrenzten Insuffizienzen ohne Sepsiszeichen kann unter Antibiotikatherapie und enger klinischer Überwachung ein konservatives Vorgehen mit Drainagetherapie ausreichend sein.

Eine retrospektive Analyse von Castro et al. zeigte, dass in ihrem Patientenkollektiv 15 % der Insuffizienzen konservativ mit alleiniger Drainagebehandlung beherrscht werden konnten, während in 69 % eine PTCD und in 16 % eine chirurgische Revision erforderlich waren [26].

### Anastomosenstenose

Ähnlich wie bei Anastomoseninsuffizienzen zeigen auch die berichteten Raten für Stenosen nach Hepatikojejunostomie eine erhebliche Variabilität. In der Literatur werden Prävalenzen zwischen 0 und 16 % angegeben, wobei die tatsächliche Rate stark von der Nachbeobachtungsdauer abhängt [6–12, 21, 27–29]. Der Großteil der Stenosen entwickelt sich innerhalb der ersten 24 Monate nach dem Eingriff [27].

Pathophysiologisch handelt es sich meist um eine narbige Schrumpfung der Anastomose infolge ischämischer Prozesse, entzündlicher Reaktionen oder einer vorausgegangenen Anastomoseninsuffizienz. Weitere bekannte Risikofaktoren sind biliäre Infektionen, Reoperationen sowie technische Faktoren wie eine zu enge oder spannungsreiche Naht, eine inadäquate Kaliberangleichung zwischen Ductus hepaticus und Jejunum oder eine unzureichende mukosale Adaptation. Studien beschreiben zudem ein erhöhtes Stenoserisiko bei benignen Grunderkrankungen, prä- oder postoperativem Gallengangsstenting, kompromittierter Durchblutung, einer Anastomose durch nicht spezialisierte Operateure sowie – am stärksten ausgeprägt – nach vorausgegangener Anastomoseninsuffizienz [27–29].

Klinisch manifestiert sich eine Stenose häufig erst Monate bis Jahre nach dem Eingriff. Leitsymptome sind rezidivierende Cholangitiden, laborchemisch nachweisbare Cholestaseparameter (erhöhtes Bilirubin, AP, γGT), unspezifische Oberbauchbeschwerden oder Fieberepisoden. In fortgeschrittenen Fällen kann es zur Entwicklung einer sekundären biliären Zirrhose kommen. Die Diagnostik erfolgt primär mittels bildgebender Verfahren wie der MRCP. Bei weiterem Verdacht oder therapeutischer Fragestellung kommen auch ERC oder PTC zum Einsatz, wobei Letztere zusätzlich eine therapeutische Intervention ermöglichen.

Therapeutisch stehen sowohl endoskopisch-interventionelle als auch chirurgische Maßnahmen zur Verfügung. Bei geeignetem Zugang kann eine perkutane Ballondilatation mit oder ohne Stenteinlage durchgeführt werden, wobei wiederholte Interventionen häufig erforderlich sind. In spezialisierten Zentren konnten damit Erfolgsraten von bis zu 96 % berichtet werden [27–29]. In Fällen therapierefraktärer oder hochgradiger Stenosen ist eine chirurgische Revision mit Neuanlage der Hepatikojejunostomie erforderlich. Hierbei sind die sorgfältige Entfernung des fibrotischen Bereichs und ggf. eine Verlängerung des Ductus hepaticus durch seitliche Erweiterungsplastiken (Hepp-Couinaud-Technik) entscheidend (Abb. 5).

## Fazit für die Praxis

Für eine erfolgreiche biliodigestive Anastomose sind spannungsfreie Naht, gute Durchblutung und präzise Mukosa-Mukosa-Adaptation essenziell. Feines, resorbierbares Nahtmaterial sollte verwendet werden. Die Hepatikojejunostomie gilt als Standardtechnik, da sie eine bessere Durchblutung und geringere Komplikationsraten als alternative Rekonstruktionen bietet. Kleine Gallengänge erfordern das Verwenden einer Lupenbrille und ggf. Erweiterungsplastiken wie die Hepp-Couinaud-Technik zur Optimierung der Naht.

Weder die Nahttechnik (Einzelknopf vs. fortlaufend) noch der operative Zugang (offen vs. minimal-invasiv) zeigen signifikante Unterschiede in der Insuffizienz- oder Stenoserate. Die Wahl sollte daher nach Erfahrung und individuellen Gegebenheiten erfolgen. Die Einhaltung grundlegender chirurgischer Prinzipien bleibt jedoch entscheidend für eine komplikationsarme und dauerhafte biliäre Rekonstruktion.
